# Recent Advances in the Chemistry of Glycoconjugate Amphiphiles

**DOI:** 10.3390/molecules23010089

**Published:** 2018-01-02

**Authors:** Laurent Latxague, Alexandra Gaubert, Philippe Barthélémy

**Affiliations:** ARNA Laboratory, Inserm U1212, CNRS UMR 5320, Université de Bordeaux, F-33000 Bordeaux, France; laurent.latxague@u-bordeaux.fr (L.L.); alexandra.gaubert@u-bordeaux.fr (A.G.)

**Keywords:** carbohydrates, glycoconjugate, amphiphiles, colloids, liposomes, gels

## Abstract

Glyconanoparticles essentially result from the (covalent or noncovalent) association of nanometer-scale objects with carbohydrates. Such glyconanoparticles can take many different forms and this mini review will focus only on soft materials (colloids, liposomes, gels etc.) with a special emphasis on glycolipid-derived nanomaterials and the chemistry involved for their synthesis. Also this contribution presents Low Molecular Weight Gels (LMWGs) stabilized by glycoconjugate amphiphiles. Such soft materials are likely to be of interest for different biomedical applications.

## 1. Introduction

Carbohydrates are found in all living systems as simple sugars, polysaccharides or glycoconjugates. Thanks to their molecular complexity and connectivity, this class of biomolecules represents the third alphabet of life beside proteins and nucleic acids [1,2]. They are involved in numerous biological processes and events, including bacterial/viral interactions, immune response, cell proliferation/differentiation, growth regulation, cell signaling, recognition, adhesion, routing etc. [3,4,5,6,7,8,9]. At the molecular level, carbohydrates interact with other molecular partners to form supramolecular complexes, which serve as markers for signal transduction. Importantly, carbohydrates fully express their molecular code in these complexes by interacting with ligands such as proteins via multivalent contacts. These cooperative interactions, named the “glyco cluster effect” [4,10], provide strong binding affinity and specificity to the carbohydrates-ligands complexes, which are required to mediate the different biochemical and/or biological processes.

Very often carbohydrates are conjugated with lipids in living systems. The resulting amphiphilic properties of the glycolipid conjugates favor their anchoring to biological membranes, where they form supramolecular assemblies through morphological changes with sugar-clustered architectures, for example [11]. Thus, biomimetic approaches have been developed to emulate natural glycolipids anchored in biological membranes [12]. In this field, many different synthetic glycoconjugate amphiphiles have been investigated, including glycoliposomes [13], glycodendrimers [14], or self-assembled monolayers (SAM) [15].

In this contribution, we will present recent advances on bioinspired glycoconjugates based amphiphiles. We wish to underline that these bioinspired molecules offer new perspectives in the fields of biomedicine or soft materials. Metal-based (Au, Ag, etc.) glyconanoparticles, glycosylated quantum dots or glycopolymers will not be addressed here. Comprehensive reviews can be found elsewere [16,17,18,19]. In the first section, the synthetic access to this class of amphiphiles is presented. Second, we summarize recent contributions on functionalized carbohydrate scaffolds. This part includes: (1) glycocyclodextrins (2) glycocalixarenes and (3) glycodendrimers. Vesicles and liposomes featuring glycoconjugates are highlighted in the third section. This article culminates in the last section with very recent developments on soft materials and in particular promising gels for biomedical applications.

## 2. Glycolipids Synthesis Overview

Glycolipids (GLs) are sugar-containing lipids and as such, are amphiphilic molecules able to self-assemble in supramolecular structures giving rise to various soft materials. Conjugation of carbohydrates to a lipid scaffold relies on a few general methods illustrated below for simple GLs (Table 1). Copper catalyzed azido-alkyne cycloaddition (CuAAC) also known as “click”-chemistry with its high chemo- and region-selectivity, its tolerance to a wide variety of solvents (including water) offers mild reaction conditions particularly useful in glycoconjugate synthesis [20]. When copper catalysis is not desirable owing to the metal cytotoxicity or its difficult elimination from the reaction medium, a strain-promoted azide-alkyne cycloaddition (SPAAC) with cyclooctynes, also termed ‘copper-free’ click-chemistry, has been developped by Bertozzi et al. [21] This strategy is based on the distorted sp-bond angles in cyclooctyne derivatives (~160° vs. 180° in a linear alkyne) which dramatically accelerates the azide-alkyne cycloaddition rate [22]. Beside CuAAC and its variants—the thiol-yne/ene click-chemistry (a radical mediated addition of thiols to alkynes) may also be appointed—a Staudinger reaction between glycosylated alkyl azides and fatty acid chlorides is also interesting (not to be confused with the Staudinger ligation, see Section 4). Finally, GLs are still often formed in accordance with standard glycosidation reactions with carbohydrate donors (thioglycosides, halogenoglycosides, peracetylated glycosides, etc.) or a combination of one of the above methods.

We will go into more details on the synthesis of more complex GLs featuring several sugar units. Some of them—that can fall in that respect into the glycocluster category—are glycosphingolipids with potent immunostimulant properties. They act as ligands of CD1d (a presentation protein of antigen-presenting cells) and the resulting binding complex is recognized by the natural killer T (NKT) cells which in turn secrete large quantities of various cytokines that mediate and regulate the immune response [28]. For example, α-Lac Cer, Gb3 and iGb3 were synthesized by the Wang [29] and Savage [30] groups (Table 2). Glycosphingolipids are found naturally in the cell membranes of almost all living organisms. Their lipidic moiety is made of sphingosine. Once acetylated by a fatty acid on their amino group, they give ceramide which in turn can be connected to one or several sugar residues on their primary hydroxyl group. The resulting GL also known as sphingolipid is called cerebroside with one Glc or Gal residue, or ganglioside when attached to an oligosaccharide featuring at least one sialic acid (*N*-acetylneuraminic acid Neu5Ac or *N*-glycosylneuraminic acid Neu 5Gc).

**Scheme 1:**
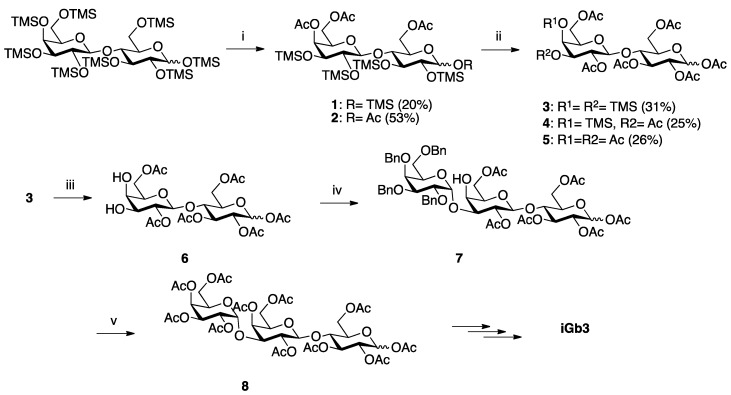
Synthesis of the saccharide moiety 8 of isoglobotriaosyl ceramide (iGb3). Reagents and Conditions: (i) Ac_2_O, Py, 3 eq. AcOH, MW, 125 °C, 1.25 h; (ii) excess Ac_2_O, Py, 3 eq. AcOH, MW, 125 °C, 1.25 h; (iii) Pd(OH)_2_/C, H_2_ 1 atm, MeOH, 0.5 h, 91%; (iv) 1-acetyl-2,3,4,6-benzyloxy-β-d-galatopyranoside, TMSI, DCM, 0 °C, 0.5 h then AgOTf, 4A MS, DCM, −78 to −30 °C, 4 h, 44%; (v) Pd(OH)_2_/C, H_2_ 1 atm, MeOH, 1 h then Ac_2_O, TEA, cat DMAP, DCM, 3 h, 85%.

Recently, the saccharide moiety **8** of isoglobotriaosyl ceramide (iGb3) was synthesized following the emergent concept of step-economy [31,32,33,34] avoiding multiple protection/deprotection steps and is therefore especially promising in glycoconjugate chemistry. In this context, the regioselective silyl exchange technology (ReSET) gives partially acetylated and silylated carbohydrates easily transformed in either glycosyl donors or acceptors [35], which greatly simplifies complex GLs synthesis (Scheme 1) [36].

Basically, per-*O*-silylated lactose undergoes selective exchange of TMS ethers for acetate protecting groups in pyridine with acetic anhydride, depending on the amount of excess AcOH added and the microwave reaction time. Compound **3** was thus obtained in two steps and selective deprotection of the silyl ethers yielded the acceptor **6**. Glycosidation with in situ prepared per-OBn galactosyl iodide with silver triflate afforded the trisaccharide **7** further acetylated to give the **iGb3** trisaccharide scaffold **8**. This new strategy, giving better yields and stereoselectivity in fewer steps, should represent an attractive alternative to more conventional methods.

**Figure 1:**
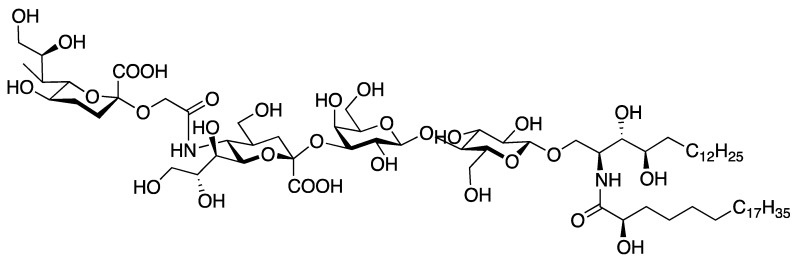
LLG-3 ganglioside structure.

Complex glycosyl ceramides, namely gangliosides, which are present in the nerve cells [37], are mainly enzymatically synthesized with the help of glycosyl transferase. For example, the starfish ganglioside **LLG-3** featuring a tetrasaccharide sphingolipid has been bioenzymatically prepared by an engineered endoglycoceramidase II glycosynthase (Figure 1).

**Figure 2:**
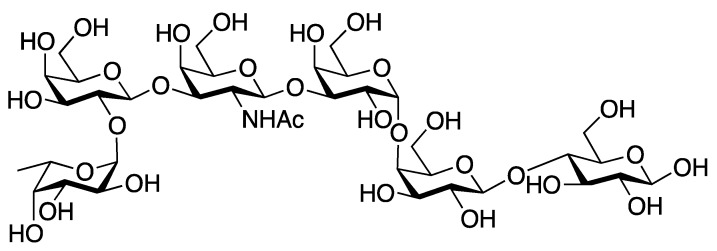
Globo-H.

Likewise, Globo-H (Figure 2), a tumor-associated antigen, has induced the development of a therapeutic vaccine based on this synthetic hexasaccharide chemically [38] or enzymatically synthesized [39].

**Scheme 2:**
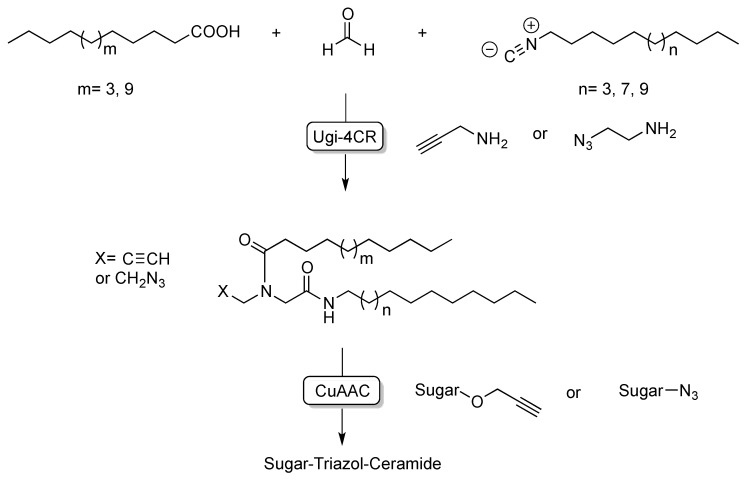
Synthetic pathway of sugar-triazol-ceramides.

Glycosphingolipids featuring various mono- and trisaccharide moieties were synthesized by a combined multicomponent/click approach [40]. The ceramide skeleton synthesis involves a fatty acid, a lipidic isocyanide, para formaldehyde and an amine (either alkynyl or azido-amine) in a one-pot Ugi-four component reaction. The resulting ceramide mimic is then conjugated with a glycosyl azide (or alkyne) through CuAAC “click” chemistry as illustrated below (Scheme 2).

This novel approach to complex GLs turned out to be very efficient with average yields of nearly 80% for both sequential steps, facilitating the rapid creation of libraries for this class of Ugi/click glycolipids.

## 3. Functionalized Carbohydrate Scaffolds

Functionalized carbohydrate scaffolds are often related to the concept of multivalency. Multivalency can be defined as the “ability of a particle (or molecule) to bind another particle (or molecule) via multiple and simultaneous non-covalent interactions” [41]. Knowing that: (1) carbohydrates are involved in a wide range of biological processes via non covalent interactions with lectins (which are specific carbohydrate proteins found inside and outside cells in animals, plants and microorganisms) [42,43,44,45]; (2) the binding affinity between carbohydrates and proteins is very weak, then, the multivalency concept a.k.a. “cluster effect” becomes perfectly obvious: multiple and simultaneous interactions occurring between lectins and their sugar ligands will result in a considerably stronger binding [46,47]. In this regard, functionalized carbohydrate scaffolds are well suited for ligand presentation as multivalent glycoconjugates. Recent reviews have described such multivalent glycoconjugates and their therapeutic efficiency [48,49]. Cyclodextrins and calixarenes, for example, offer intrinsic scaffolds for this purpose, owing to their numerous functionalization opportunities, whereas dendrimers can be tailored to the desired generation and functionality.

### 3.1. Glycocyclodextrins

Cyclodextrins (CDs) are torus-like cyclic oligosaccharides, α-, β- and γ-CD being the most well-known of them. Thus, α-CD is composed of six glucopyranose units linked via an α-1,4 glycosidic bond, β-CD is the most often used and comprises seven Glc units compared to eight units for the larger γ-CD [50]. Their internal cavity is hydrophobic and CDs can form inclusion complexes with guest molecules.

**Scheme 3:**
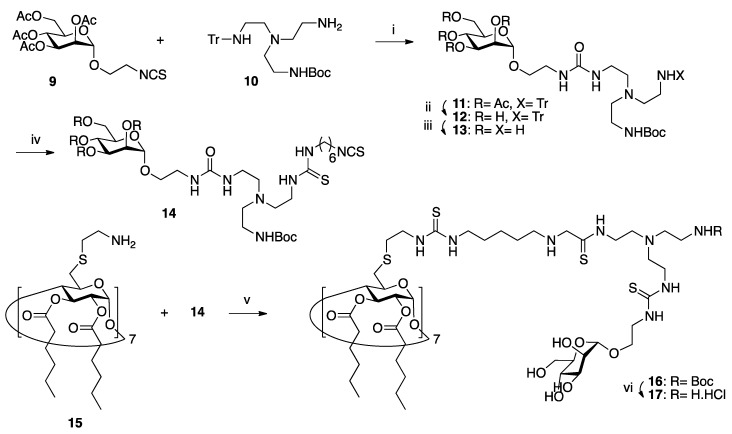
Synthetic pathway of a multivalent polycationic glyco-amphiphile cyclodextrin. Reagents and Conditions: (i) DCM, TEA, 98%; (ii) NaOMe/MeOH, 97%; (iii) 2% TFA, 99%; (iv) SCN(CH_2_)_6_NCS, acetone-H_2_O, 52%; (v) DMF, TEA, 40 °C, 48 h, 84%; (vi) TFA-DCM, r.t., 3 h, freeze-drying from diluted HCl, 99%.

A β-cyclodextrin scaffold has been used to synthesize a multivalent polycationic glyco-amphiphile cyclodextrin (pGaCD) as a gene delivery system [51,52]. The upper rim of the CD is functionalized with a glyco-cationic moiety while the lower rim exhibits lipophilic tails (Scheme 3).

Starting from the known 2-azidoethyl peracetylated-α-d-mannopyranoside and bis(2-aminoethyl)-(2-*N*-BOC-ethyl)amine respectively, the isocyanato sugar derivative **9** and the orthogonally protected **10** were readily obtained. They were reacted with TEA to afford the adduct **11** in almost quantitative yield. Acetyl and then trityl cleavage were conducted with the same efficiency to give the amine **13** subsequently reacted with the spacer 1,6-hexamethylene diisocyanate to afford the glyco-cationic moiety **14**. A thiourea linkage between the known β-CD derivative **15** and compound **14** was formed under TEA catalysis, and carbamate deprotection gave the final pGaCD **17** in excellent yield. Here, the chemistry involved in this synthesis uses an amine-isothiocyanate reaction for the high yields it delivers and to provide a thiourea belt, which is expected to interact favorably with the phosphate groups of the plasmid chain. This pGaCD was indeed able to self-assemble in the presence of plasmid DNA leading to stable nanoparticles ranging from 70 to 150 nm in size.

### 3.2. Glycocalixarenes

Calix[*n*]arenes are macrocyclic compounds composed of n phenolic units connected by methylene bridges [53]. They possess a vase shape with a cylindrical hydrophobic cavity able to accomodate inclusion compounds much like CDs. Both sides of the calixarene (upper and lower rim) can be easily derivatized—for what we are concerned here—with saccharide units giving rise to glycocalixarenes [54].

**Scheme 4:**
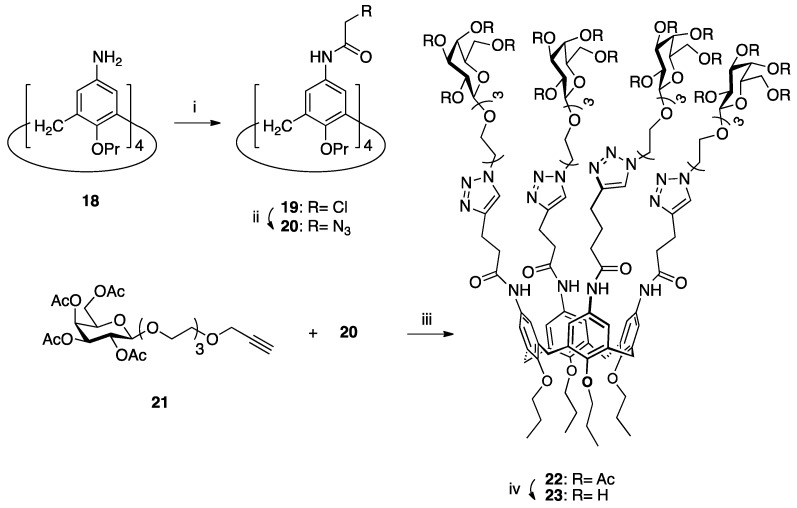
Synthesis of glycosyl calix[4]arenes from tetraamino calixarenes. Reagents and Conditions: (i) ClC(O)CH_2_Cl, DIPEA, DCM, room temperature, 6 h (55%); (ii) NaN_3_, MeOH/DMF, reflux, 1 h (75%); (iii) CuSO_4_.5H_2_O, Na-ascorbate, DMF/H_2_O, MW (150 W), 80 °C, 20 min (81%); (iv) NaOMe/MeOH, room temperature, 1 h (quantitative).

A typical example is the synthesis of galactosyl- and lactosylcalix[4]arenes from tetraamino calixarenes with a CuAAC coupling as the key-step (Scheme 4) [55].

Calixarene derivative **18** was first treated with chloroacetylchloride and then with sodium azide to give the tetraazido calixarene **20**. Peracetylated galactopyranose was reacted with monopropargyl triethylene glycol in the presence of BF_3_·OEt_2_ to yield the azidosugar derivative **21**. Coupling the later compound with **20** under click-chemistry conditions yielded **22** further deacetylated to the final calixarene **23**. The lactosyl cluster derivative was similarly prepared. An interesting feature with calixarenes is their relative mobility from a conformational viewpoint. Depending on the size of the alkyl substituents on the phenolic OH lower rim, they can actually exist in four main conformations (the so-called cone, partial cone, 1,2-alternate and 1,3-alternate) [56]. In this regard, the 1,3-alternate conformation was also prepared with both saccharides by the authors. Surface plasmon resonance (SPR) experiments were then carried out on surface immobilized His-tagged Galectin-3 showing that a better affinity was observed with the lactosyl cluster and the cone conformation over the 1,3-alternate one.

### 3.3. Glycodendrimers

Glycodendrimers are currently emerging as an area of growing activity as multivalent glycoclusters as evidenced by the numerous surveys recently dedicated to this field (see reference [49] for the most recent review).

**Scheme 5:**
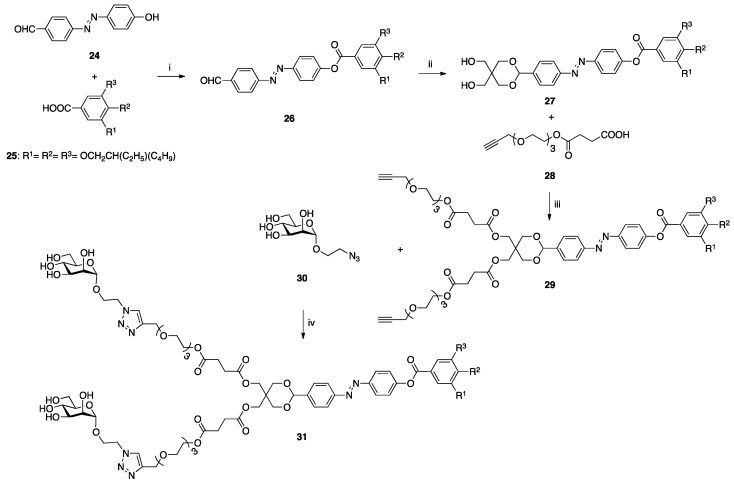
Synthesis of a d-mannose dendrimer amphiphile Reagents and Conditions: (i) 4-(dimethylamino)pyridinium *p*-toluenesulfonate, DCC, DCM, 56–61%; (ii) pentaerythritol, PTSA, DMF, 50 °C, 46–61%; (iii) 4-(dimethylamino)pyridinium *p*-toluenesulfonate, DCC, DCM, 46–67%; (iv) CuSO_4_, sodium ascorbate, THF/water (2:1 *v*/*v*), 40 °C, 31–54%.

In a recent contribution [57], a library of 12 amphiphilic Janus glycodendrimers [58] composed of variable carbohydrate head groups and hydrophobic tail groups linked to an azobenzene core have been synthesized using successive Steglich esterification and Hüisgen cycloaddition reactions. The general synthesis is outlined below for one of the dendrimers **31** bearing branched alkyl chains on one side and a d-mannose derivative on the opposite side (Scheme 5).

The resulting glycodendrimers self-assembled in water to give uniform cylindrical micelles of various size as evidenced by differential light scattering (DLS) and small angle neutron scattering. The azo-benzene core exhibiting trans-cis isomerism under UV-light, was used here to make the glycodendrimer micelles phototunable, allowing to evaluate their potency as inhibitors of LecA and LecB bacterial lectins. Only a moderate activity was recorded, though, a result imputed to a low light penetration inside the micelle to reach its core.

Chevolot, Morvan et al. targeted the same Lectin A with a family of 32 glycodendrimers of generation 0 and 1 constructed by CuAAC coupling with azido-functionalized glycosides [59]. High affinity values were obtained when aromatic aglycones were used with the carbohydrate motif, confirming that the topology of the glycocluster is equally important as multivalency itself.

**Scheme 6:**
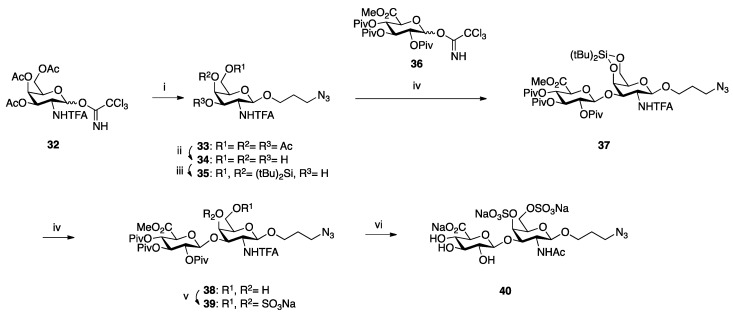
Synthesis of a dissacharide unit starting from trichloroacetimidate **32**. Reagents and Conditions: (i) 3-Azidopropanol, TMSOTf, DCM, 0 °C, 81%; (ii) NaOMe, MeOH, 96%; (iii) tBu_2_Si(OTf)_2_, Py, 94%; (iv) TMSOTf, DCM, 0 °C, 80%; (v) (HF)n·Py, THF, 0 °C, 95%; (vi) SO_3_·Me_3_N, DMF, 100 °C, MW, 85%; (vi) LiOH, H_2_O_2_, THF; NaOH, MeOH/H_2_O; Ac_2_O, Et_3_N, MeOH/H_2_O, 73%.

Interestingly, multivalency of small saccharide units can also be used to mimic the effect of larger polysaccharides. Chondroitin sulfate (CS) for example, is a heterogeneous polysaccharide involved in several diseases. CS binds to many proteins via the disaccharide GlcA-GalNAc (4,6-di-OSO_3_) unit. The need for homogeneous synthetic CS is thus clear but its synthesis is challenging. Therefore, glycodendrimers featuring the sulfated disaccharide group may be a good alternative for this purpose. The dissacharide unit synthesis begins with the trichloroacetimidate **32** treated with 3-azidopropanol and TMSOTf as promoter (Scheme 6). Compound **6** was then protected in two steps with a 4,6-*O*-di-*tert*-butylsilylene group leaving the 3-OH free for glycosidation with the donor **36**.

**Scheme 7:**
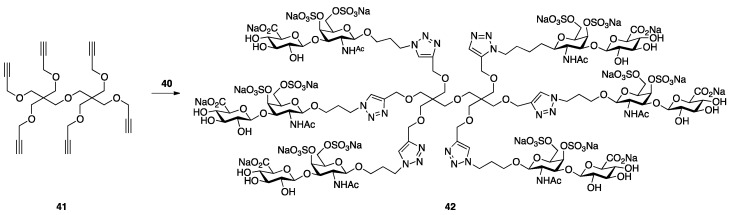
Synthesis of a hexavalent glycodendrimer via CuAAC coupling Reagents and Conditions: CuSO_4_·5H_2_O, sodium ascorbate, TBTA, DMSO/PBS buffer, room temperature, overnight, quantitative.

The disaccharide **37** was then selectively deprotected on its 4- and 6-OH positions to allow their subsequent sulfonation using a large excess of sulfur trioxide trimethylamine, to eventually afford the desired compound **40** after basic hydrolysis and deacetylation. Tri-, tetra- and hexavalent dendritic cores were then reacted with the disaccharide units under CuAAC conditions illustrated below for the hexavalent glycodendrimer **41** (Scheme 7).

All glycodendrimers were then successfully investigated for their multivalent properties against midkine (an heparin-binding growth factor) to give IC_50_ up to 1.2 µM for the hexavalent compound **42**. On the other hand, the disaccharide itself exhibited a very low affinity for midkine (250 µM) confirming the value of the glucomimetics approach developped in this work.

Many glucodendrimer syntheses take advantage of the CuAAC coupling [60,61,62], and not only for connecting the saccharide moiety. Thus, Das and Mukhopadhyay [63] for example, synthesized a propargyl-functionalized Zn-porphyrin, further conjugated with the separately constructed azido-functionalized mannose dendritic building blocks. Synthesis of the 1st and 2nd generation carbohydrate dendrimers involved a click reaction, as well as the coupling step with the porphyrin core.

Finally, thiol-yne coupling (TYC) or thiol-ene coupling (TEC) are mild and high-yielding reactions which proved especially useful in glycosidation synthesis and this topic has recently been reviewed [64].

## 4. Vesicles, Liposomes

It is known that the overall shape of an aggregate is driven by the shape of the amphiphilic molecule that produced it. When the amphiphiles are wedge-shape (cross section of the polar head is greater than that of the side chain—a typical situation for a single lipidic chain surfactant) their self-association produces a spherical micellar structure. For truncated-cone shapes, aggregation gives cylindrical micelles. Finally, for cylindrical amphiphiles (cross-section of the polar head roughly equals that of the side-chain, usually when a double fatty acid chain is involved) a planar bilayer sheet is formed. Then, closure of the bilayer sheet yields spherical structures called vesicles, entrapping an aqueous domain isolated from the outer aqueous medium. Artificially constructed vesicles are called liposomes. Depending on experimental conditions, several types of liposomes are obtained: multilamellar vesicles (MLV), small unilamellar vesicles (SUV), large unilamellar vesicles (LUV) or giant unilamellar vesicles (GUV) [65,66,67].

**Scheme 8:**
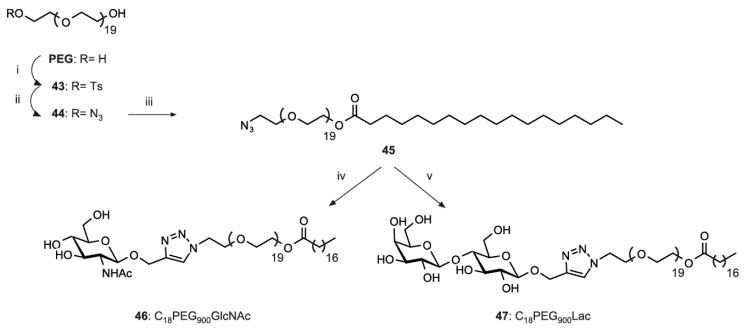
Synthesis of stearoyl amphiphiles, GlcNAc **46** and Lac **47**. Reagents and Conditions: (i) TsCl, Ag_2_O, KI, DCM, 55%; (ii) NaN_3_, DMF, 85%; (iii) DCC, DMAP, stearic acid, DCM, 82%; (iv) CuSO_4_, sodium ascorbate, propargyl-2-*N*-acetamido-2-deoxy-β-d-glucopyranoside, THF/H_2_O, 72%; (v) CuSO_4_, sodium ascorbate, propargyl-β-lactopyranoside, THF/H_2_O, 67%.

Spherical micelles have been obtained from the self-assembly in water of a series of macromolecular glycosylated amphiphiles. They have been prepared by CuAAC coupling of 1-*O*-propargyl saccharides (*N*-acetyl glucosamine and lactose) and various azido-terminated PEG900 esters [68,69,70,71]. In a representative work, C_18_PEG_900_GlcNAc **46** and C_18_PEG_900_Lac **47** were obtained from a common intermediate **45** (Scheme 8). This stearate derivative was prepared in a few steps from PEG900 by monotosylation followed by substitution of the tosyl group with sodium azide.

**Figure 3:**
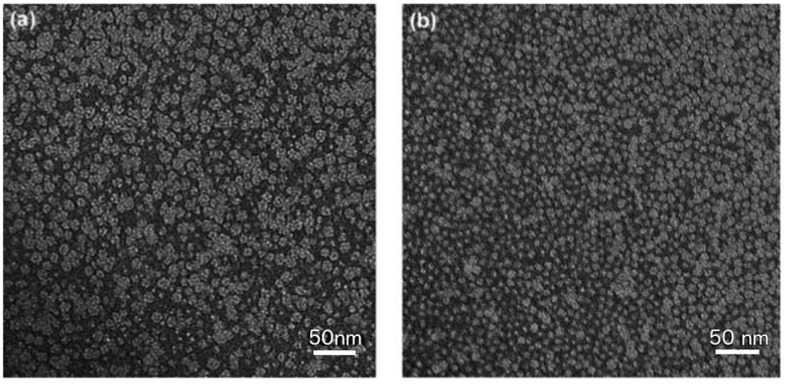
Transmission electronic microscopy (TEM) images for the self-assemblies of **46** (**a**) and **47** (**b**) (taken with permission from reference [69]).

Both glycoconjugates **46** and **47** spontaneously self-assembled in water into spherical micelles with a mean diameter of 10 nm (Figure 3).

Specific interactions of both **46** and **47** with lectins (Wheat Germ Agglutinin and Peanut Agglutinin) proved the presence of the sugar residues at the surface of the micelles demonstrating their potential application in drug delivery mediated by carbohydrate-protein interactions.

**Scheme 9:**
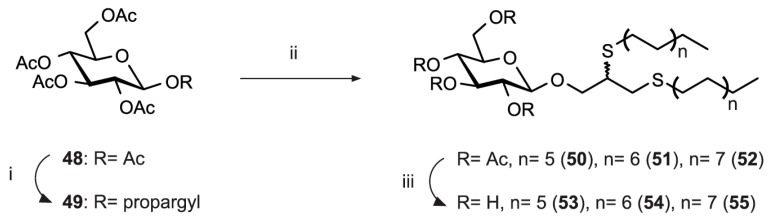
Thiol-yne/ene chemistry for the synthesis of GLs. Reagents and Conditions: (i) Propargyl alcohol, BF_3_·OEt_2_, DCM, 0 °C to room temperature, 2 h, 91%; (ii) CH_3_CH_2_(CH_2_CH_2_)_n_SH, AIBN, dioxane, 100 °C, 1 h, 95%; (iii) MeONa, MeOH, room temperature, 1 h, 96%.

To efficiently link a sugar derivative to a lipidic tail, thiol-yne/ene click-chemistry already quoted in Section 3.3) can be considered as a complementary tool to the Hüisgen (CuAAC) reaction [72]. In a recent publication, Goyard et al. [24] reacted 1-propargyl-2,3,4,6-tetra-*O*-acetyl-β-d-glucopyranoside **49** with dodecane-, tetradecane- and hexadecanethiols in the presence of AIBN to give the expected GL derivatives (as 1:1 diastereoisomer mixtures) in very high yields (Scheme 9).

The Gal (**56**), Man (**57**) and Lac (**58**) derivatives have been prepared similarly. The GLs were capable of self-assembling into vesicles. Among them, vesicles prepared from **53** and **57** formed multivalent interactions with concanavalin A (ConA) used as a model lectin. Furthermore, **57** was able to bind to murin dendritic cells and to encapsulate hydrophobic compounds like Taxol, thus paving the way to the vectorization of active substances to specific cells.

A method of choice for the surface functionalization of liposomes calls on the Staudinger ligation [73] named after the well-known Staudinger reaction between a phosphine and an azide giving an iminophosphorane which hydrolizes into an amine [74]. Here, to avoid the iminophosphorane hydrolysis, the phosphine features an electrophilic trap such as a methyl ester producing an amide bond (Table 3) [75,76].

**Scheme 10:**
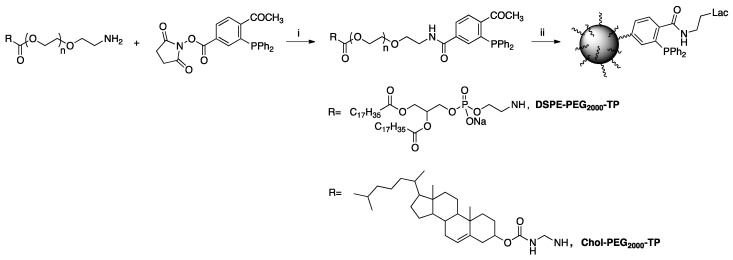
Chemically-selective glycol-functionalization of liposomes by Staudinger ligation. Reagents and Conditions: (i) DCM, TEA (50% Chol-PEG_2000_-TP, 30% DSPE-PEG_2000_-TP); (ii) Liposome formulation then Staudinger ligation with 1-(2-azido)ethyl lactopyranoside.

The chemically-selective surface glyco-functionalization of liposomes through Staudinger ligation was popularized by Sun and co-workers [78,79,80]. They recently evaluated this reaction with a lactosyl azide and different ratios of two types of anchoring lipids in DPPC liposomes [81]. Lipids were synthesized from cholesterol-PEG_2000_-NH_2_ and commercially available DSPE-PEG_2000_-NH_2_ with 3-diphenylphosphino-4-methoxycarbonylbenzoic acid active ester (Scheme 10) to give functional vesicles with respect to their stability, encapsulation and release capacity, and their lectin binding efficiency.

## 5. Gels

Hydrogels are found in our everyday life in a variety of forms and products. For example hydrated gel materials can be used as cosmetics (shampoo, hair gel, soaps etc.). Importantly most of the gels, if not all, are derived from polymers. This type of gels has been known for many years with different applications in the fields of materials science, medicine, cosmetics, etc. Low molecular weight gels (LMWGs), which were developed more recently, offer an interesting alternative to polymer-based gels. Indeed, thanks to their supramolecular nature LMWGs are thermoreversible and allow a rapid response to external stimuli. Also, LMWGs can be used as biodegradable systems because of an easier clearance and/or elimination from the body compared to regular polymeric gels. Nevertheless, the supramolecular assemblies obtained using LMWGs are still poorly controlled and it is still difficult to properly predict the gel behaviours depending on the molecular structure of the components. In this regard, several LMWG based bioconjugates have been investigated recently for their supramolecular gel properties, including lipids, carbohydrates, nucleosides, amino acids or peptide derivatives. Among them, glycoconjugates have been discovered to be interesting gelators for biomedical applications. In this section, we present several examples of synthetic sugar based amphiphiles forming LMWGs.

A gel can be defined as a molecular network able to immobilize a fluid (organic solvent or water) in a very large amount (up to more than 99% *w*/*w*) giving rise to a soft, jelly-like material [82,83]. Such material can be made of polymeric chains or from the self-assembly of small molecules (a.k.a. low molecular weight gelators) a process driven by weak molecular interactions [84]. It is commonly believed now that gelation is a stepwise process, starting from the molecular level to the macroscopic structure. First, non-covalent interactions (H-bonding, π-π stacking, hydrophobic forces) must take place between gelling molecules, leading to their nanostructuration in a one-dimensional fashion (fibers formation). Then, the growing fibers form a continuous three-dimensional entangled network in the solvent under the same non-covalent set of forces, to eventually give rise to a macroscopic soft material, i.e., a gel. The exact mechanism of self-organization of the gelling agents is still poorly understood especially in water. Nevertheless, a simple packing model to mimic fiber formation during self-assembly was recently developed [85]. The fiber is considered as the stacking of discrete prisms exhibiting various hydrophobic and hydrophilic faces corrected with several weighting coefficients representing free energy penalties. Use of this model demonstrated that for selected classes of LMWGs, the fiber structure represents the thermodynamic minimum.

### 5.1. Glycoconjugate Based Gels

Hydrogels possess many applications in tissue engineering [86,87], biosensing, drug or gene delivery [88,89,90], water depollution [91], etc. The most relevant gelling agents for biomedical applications are biologically-inspired molecules such as peptide-amphiphiles, oligonucleotide-amphiphiles or glycosylated-amphiphiles. In the context of this survey, only low molecular weight glycosylated-amphiphiles will be addressed with a particular interest in glyconucleolipid amphiphiles.

**Scheme 11:**
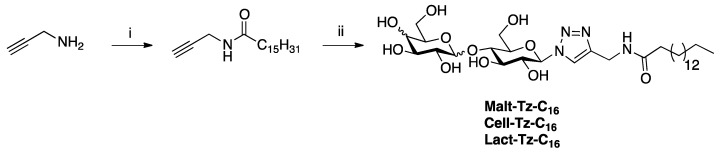
Synthesis of lipidic disaccharides by CuAAC cycloaddition Reagents and Conditions: (i) C_15_H_31_CO_2_H, THF, DCC/HOBt, room temperature, 2 days, 75%; (ii) Malt(OAc)_7_-N_3_ or Cell(OAc)_7_-N_3_ or Lact(OAc)_7_-N_3_, CuBr, PMDETA, 87%, 80% and 81% resp. then MeONa, MeOH, 85–90%, 77% and 80% resp.

For example, monolipidic disaccharides have been prepared by CuAAC cycloaddition of the corresponding sugar azides with *N*-propargylpalmytoyl amide (Scheme 11) [92,93].

**Figure 4:**
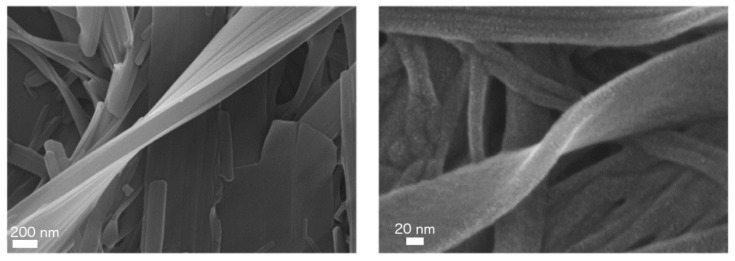
FESEM images of a single ribbon of Cell-Tz-C16 (0.5 wt % water) xerogel on the left and of Lact-Tz-C16 on the right (1 wt % water) (taken with permission from reference [93]).

The resulting disaccharidic GLs were efficient hydrogelators (0.5 to 1 wt %). The contribution of the triazole ring (giving π-π stacking) and the amide-NH moiety (H-bonding) in the hydrogelation process was evidenced by nuclear magnetic resonance (NMR). Furthermore, field emission scanning electron microscopy (FESEM) images of a single ribbon of Cell-Tz-C_16_ exhibited a right-handed twist whereas Lact-Tz-C_16_ showed a left-handed twist suggesting that the nature of the disaccharide polar head influences the supramolecular chirality (Figure 4).

Another aspect of GLs is their ability to self-aggregate with a long-range molecular ordering giving rise to a crystal-like structure while retaining a liquid behaviour. This state of matter is the distinctive feature of liquid crystals (LCs). Thus, simple GLs regarded as rod-like molecules self-aggregate into lamellar assemblies with a tendancy to form columnar or spherical structures according to their tail to head volume ratio [94]. This phase change can be induced by an increase in temperature (thermotropic LCs) but also if their concentration is varied in a solvent (lyotropic LCs) resulting in more mobile structures giving rise to additional phase changes. At temperatures above 90 °C, Malt-Tz-C_16_ in the previous example exhibits a LC behaviour. For Cell-Tz-C_16_ and Lact-Tz-C_16_ the liquid crystalline phases (mesophases) appeared above 150 °C, and Smectic A phase (molecules are directionally ordered into layers) for the latter compounds was evidenced by X-ray diffraction. Of course, dealing with LC properties of GLs is beyond the scope of this account and further reading and examples can be found elsewhere [95,96,97]. Note, however, that thermotropic LC phases were much less studied than LC structures formed in water, and other biomolecules can form such materials [98].

**Scheme 12:**
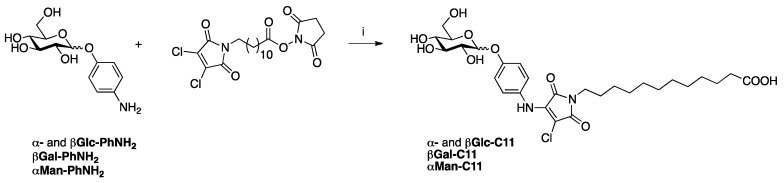
Synthesis of asymmetric glycol bola-amphiphiles. Reagents and Conditions: (i) DIEA, DMF, room temperature, 24 h, 64% **βGlc-C11**, **αGlc-C11** and **βGal-C11**, 80% **αMan-C11**.

Original asymmetric glyco bola-amphiphiles were obtained by Ochi et al. [99] by reacting 4-aminophenylgluco-, galacto- and manno- pyranosides with an aminododecanoic acid derivative (Scheme 12). Despite the presence of an activated ester at one end of the hydrocarbon chain, chlorine displacement on the maleimide moiety unexpectedly occurred with good yields.

All bolaform GLs turned out to be excellent hydrogelators with a critical gelation concentration (CGC) < 0.1 wt %. Interestingly, the π-conjugated 2-anilino-3-chloromaleimide is a known chromophore with a λ_max_ value appearing near 400 nm. As a result, the chloromaleimide GLs exhibited color change upon gelation from warm orange (sol) to yellow (gel). Furthermore, the color change was also observed upon gel incubation with the corresponding glycosidases, thanks to the proximity of the chromophore with the glycoside moiety. The enzyme selectivity is therefore retained in the gel state and makes these gels usable as sensing materials to detect an enzymatic activity by the naked eyes.

### 5.2. Mechanical Characterization of Gels

The increasing number of supramolecular gels require a characterization at the microscopic and the macroscopic levels. Specific information about the structure and dynamics of the self-assembling will be obtained. Gels are not only molecules solvated in water, oil or solvent, for hydrogels, oleogels or organogels respectively, but also secondary and tertiary structures. These structures responsible of the gelation phenomenom need to be highlighted, especially the number, type and strength of the interactions. One way to investigate the viscoelactic behaviour of such material is rheology. This technique analyses samples at their native state enabling a better understanding of the gel macroscopic organization. Different types of setup are available for rheological studies, cone and plate systems, parallel plates and concentric cylinders, depending of the material viscosity. In each case, thin layer of gel will be spread between the movable and the stationary plates. The response of the supramolecular gels to applied oscillatory stress quantifies its viscoelastic properties by three major variables: G* (complex modulus), G’ (storage or elastic modulus) and G” (loss or viscosity modulus). The evolution of theses variables function of the imposed stress, the frequency, the time or the temperature will contribute to the characterization of gelation key points (i.e., linear viscoelastic region (LVER), strength of the gel, thixotropic behaviour, T_gelsol_, etc.) [82,83]. Almost all mechanical properties are determined within the LVER. In this domain, the evolution of moduli will be independent of the magnitude of imposed stress or strain [100]. So the macroscopic behaviour observed will only be due to the variant factors. When this factor is the temperature, the most often reported parameter will be determined: temperature of gelation (T_gelsol_). During the experiment, the temperature increases and the macroscopic properties of the supramolecular gel evolve until a break of the tertiary structure. The point when the sample goes from a gel-like state to a liquid-like state is called T_gel_ or T_gelsol_ [82,83]. Another rheological property determines for supramolecular system, especially gel composed of glycolipids, is the thixotropic behaviour. This unique behaviour is investigated by a time dependent experiment. Supramolecular assembly undergoes a mechanically stimulus leading to a collapse of the tertiary structure and to a gel-sol transition. When the external force is withdrawn, the supramolecular gel can rebuilt to its initial state (sol-gel transition). The reversibility of the system is called thixotropy. This phenomenon is essentially due to modifications at the macroscopic level without chemical alterations. Supramolecular assemblies are essentially based on weak interactions (π stacking, hydrophobic effect, H bonding) which can be disrupted and can recover from external stress enabling this thixotropic behaviour.

All these rheological parameters are crucial for the characterization of gels and is unambiguously required. The strength of gels could have an impact on its application, for example in the biomedical field allowing cell proliferation and differentiation [101,102,103]. Supramolecular assemblies are often compared to polymeric gels on various criteria and especially mechanical strength. Mei et al. [83] classify hydrogels in two categories: the polymeric and the supramolecular one. Among the polymeric classes, they distinguish the polymeric hydrogels based on covalent crosslink (Type I) or on noncovalent crosslink (Type II). The hydrogels are compared function of numerous criteria and especially the mechanical strength. Polymeric hydrogels are characterized by strong mechanical strength (extremely strong for Type I/relatively strong for type II) and supramolecular systems by weak mechanical strength.

**Figure 5:**
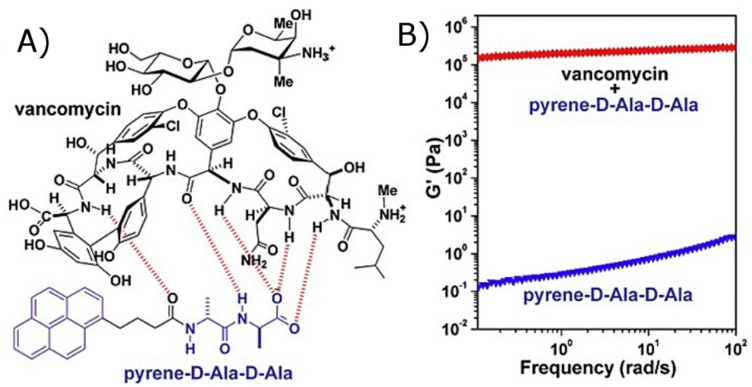
(**A**) Structure of the complex Vancomycin—pyrene d-Ala-d-Ala contained in the self-assembling system; (**B**) Evolution of the elastic modulus (G’) function of the frequency for the hydrogel with or without vancomycin (taken with permission from [86,104]).

However, the mechanical properties of a supramolecular system could be improved by the addition to the biomaterial of a ligand. Zhang et al. [104] incorporated vancomycin in a supramolecular hydrogel of self-assembled pyrene-d-Ala-d-Ala resulting in a 10^6^ fold increase of the elastic modulus (Figure 5).

Another way to improve the mechanical strength of supramolecular gels is the synthesis of new generations of low molecular weight hydrogelators which present high elastic modulus up to 10^4^–10^5^ Pa [105,106,107,108].

The molecules used to obtain such results are glycolipids based. Full rheological data about supramolecular assemblies composed of glycoconjugates are not always found but is very useful for the characterization of glycolipids based gels. The rheological data of self-assembled glycoconjugate based gels are summarized in Table 4.

Except for the molecule developed by Prasad et al. [107], the glycolipids are solvated in water allowing potential applications in the medical field. They are all prepared with low concentrations of gelator molecules (<10% (*w*/*w*)) compared to the polymeric gels. Fitremann et al. [109] developed a weak hydrogel presenting a low biocompatibility for neural cell culture. It’s a first approach for this generation of glycolipids, which will need tuning to improve the biocompatibility but also the T*_gelsol_* for biomedical applications. Barthélémy’s group found a way to design specific glycolipids allowing biomedical applications and personalization of the biomaterial with various mechanical strengths. The incorporation of a nucleoside in the glycolipid structure add a coding information and these inspired molecules present high cytocompatibility [101,102]. The glyconucleolipid is the basic structure and different generations were synthetized from this skeleton: Glycosyl-Nucleoside Fluorinated amphiphiles (GNF) [101,102,110,111] and also Glycosyl-Nucleoside Bola-Amphiphiles (GNBA) [103,105,106]. Each hydrogel obtained with this glyconucleolipid structure presents specific rheological parameters, which were used to tune the molecule design. This tuning was done to improve the mechanical strength essentially to widen the biomedical applications (scaffold for stem cells, osteoblastic cells, etc.) [102,103,104,107]. These modifications could also affect other parameters such as the T_gelsol_. Indeed, for the GNBA carbamate, an increase of the mechanical strength was observed compared to the previously synthetized GNBA [105] but also a decrease of the T_gelsol_ (36.3 °C) preventing biomedical applications for the moment. That’s why the association of organic chemistry and physico-chemical characterization (rheology) is essential to guide the design of new glycolipid molecules able to self-assemble.

The viscoelastic behaviour could also be analyzed by indentation and not only rheology. Ikeda et al. used atomic force microscopy (AFM) indentation to characterize the elastic modulus of their strong hydrogel for an application on prostate cancer cells [108].

Glycoconjugate are not only forming gel in water but also in various solvents and vegetal oils. The organogel obtain in cyclohexane exhibits a mechanical strength comparable to polymeric gels at very low concentrations (0.6% (*w*/*w*)) [107]. Even if no real applications are proposed for this new LMWG, it is a very promising new way to obtain glycolipids from renewable resources. The gelification of such molecules in vegetable oils open the way to new applications in the pharmaceutical and cosmetic fields for example.

The rheological parameters of a gel are very important for the potential biological applications: scaffold for cell’s culture, drug delivery systems, and regenerative medicine. Moreover with the examples presented here, the impact of the chemical structure on the LMWGs is clearly identified. Rheological characterization need to be compulsory for supramolecular gels allowing molecular screening and fine-tuning to adapt the chemical structure to the application.

## 6. Conclusions

Despite the substantial progress in the synthesis of soft materials, i.e., the formation of supramolecular gels with low molecular weight gelators, there is still a significant need to design, synthesize, and evaluate new glycoconjugate amphiphiles for an access to biomaterials possessing the required properties for different biomedical fields, including regenerative medicine, drug delivery or tissue engineering. Much of this activity is mainly driven by the current weak viscoelastic properties of the synthetic amphiphile gelators as well as the lack of biocompatibility for in vivo applications. With this in mind, polymer-free biomaterials that can overcome these two major hurdles are highly sought after. It is clear that the modulation of the elastic modulus (G’) and the thixotropy propreties are important limiting parameters of supramolecular biomaterials. Therefore, the design of glycoconjugate that self-assemble in a controlled manner would provide new biomaterials for constructing sophisticated supramolecular gels.

Although the control of supramolecular gel properties is still under investigation, it is important to avoid inflammatory response once this type of bioinspired material is injected or implanted in vivo. Nevertheless the chemistry of glycoconjugate amphiphiles has much to offer to the vast domain of biomedicine. Indeed, playing with the chemical diversity of the sugar code can modulate and/or trigger biological responses. The role of synthetic carbohydrate chemistry for designing and synthesizing carbohydrate derivatives featuring biological properties can be illustrated in the context of vaccines with two recent reports. In a recent publication Gauthier et al. hypothesized that the chemical modification of lipopolysaccharide (LPS)-based vaccines antigen could have a strong impact on immune responses [112]. To validate this hypothesis they prepared several oligosaccharides with the aim of deciphering the immunogenic epitopes of surface polysaccharides. They demonstrated that synthetic chemistry and sugar modifications were crucial for recognition. In another recent report Guler et al. investigated the physico and biological properties of novel mannosylated glycopeptides. Interestingly, the nanofibers resulting from the self-assemblies of these amphiphiles were well adapted for the delivery of immunogenic mimetic antigens [113].

The future development of the glycoconjugate amphiphiles, in particular the synthetic access to a large diversity of molecular and supramolecular structures, will certainly witness exciting discoveries in different biomedical areas, including biomaterials, medicinal chemistry or vaccination.

## Figures and Tables

**Table 1:** Some illustrative synthetic strategies for GL formation. More detailed examples will be discussed in the following paragraphs.

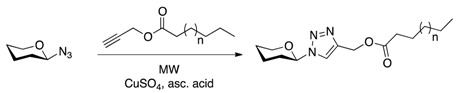	[23]	CuAAC click-chemistry
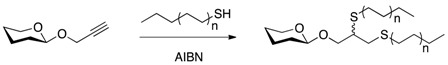	[24]	Thiol-yne click chemistry
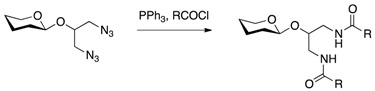	[25]	Staudinger reaction + activated carbonyl
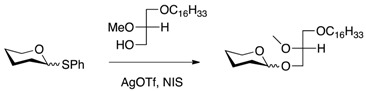	[26]	Thioglycosidation method
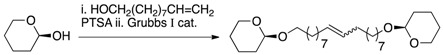	[27]	Fisher glycosidation + metathesis

**Table 2:** Examples of sphingolipids with α-Lac Cer, α-Gb3 and α-iGb3.

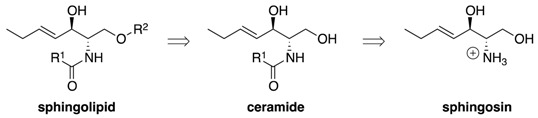
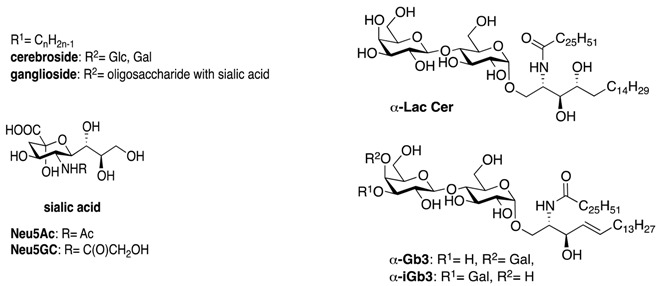

**Table 3:** Mechanisms for the Staudinger reaction and ligation in water. Note that the Staudinger ligation leaves a diphenylphosphine residue in the molecule. The “traceless” Staudinger ligation (especially useful in peptide synthesis) [77] is not depicted here.

**Staudinger reduction**
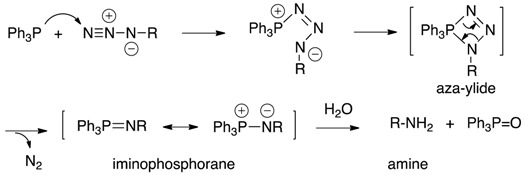
**Staudinger ligation**
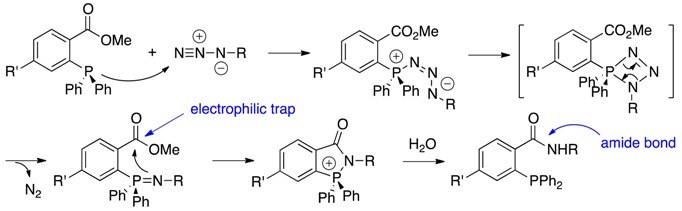

**Table 4:** Rheological characterization of low molecular weight hydrogelators self assembled of glycolipids and their applications.

Solvent	Concentration % (*w*/*w*)	Morphological Structure	G’ (Pa)	Thixotropic Behaviour	T_gelsol_ (°C)	Applications	Ref.
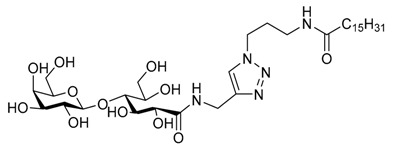							
Water	2	Wormlike micelles	≈500	Yes	29	Reduced biocompatibility	[109]
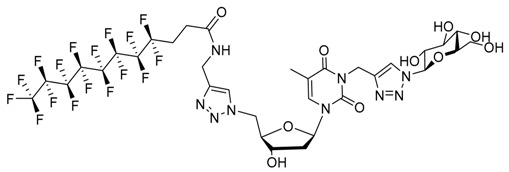							
Water	1.5	Nanofibers	800	Yes	52	Matrix for cell culture Biomaterial Decontamination	[101,102,110,111]
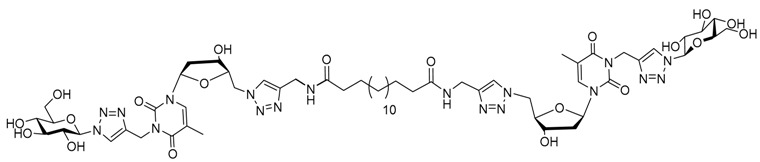							
Water	11.55 mM	Nanofibers	5 × 10^3^	Yes	62	Injection in mice Biomaterial	[106]
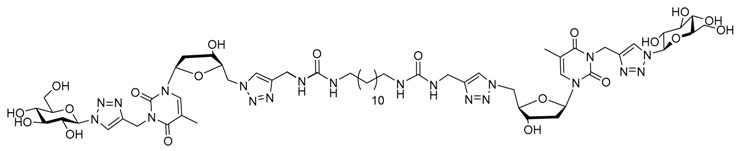							
Water	11.55 mM	Nanofibers	12 × 10^3^	Yes	79	Injection in mice No foreign body reaction	[106]
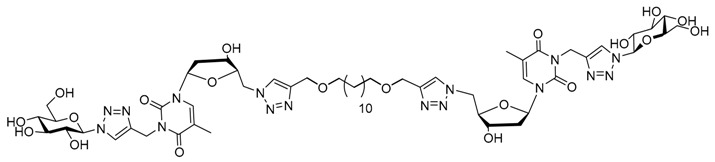							
Water	2	Nanofibers	30 × 10^3^	Yes	46	Scaffold for stem cells	[103]
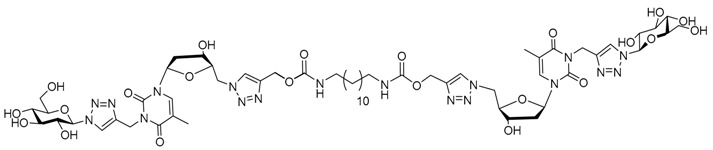							
Water	4	Nanofibers	57 × 10^3^	Yes	36	No biomedical application for the moment	[105]
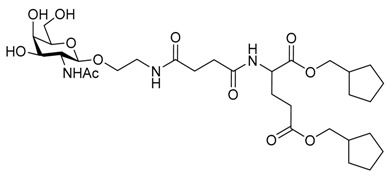							
Water	5	Nanofibers	63 × 10^3^	-	-	Enzyme/cell responsive capsule	[108]
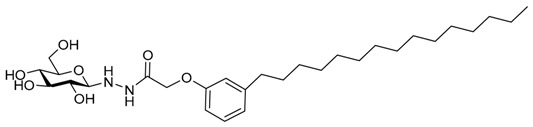							
Cyclohexane	0.6	Nanofibers	100 × 10^3^	-	49	No application for the moment	[107]
